# Evaluating adherence to recommended diets in adults 1991–2015: revised China dietary guidelines index

**DOI:** 10.1186/s12937-019-0498-3

**Published:** 2019-11-11

**Authors:** Feifei Huang, Zhihong Wang, Liusen Wang, Huijun Wang, Jiguo Zhang, Wenwen Du, Chang Su, Xiaofang Jia, Yifei Ouyang, Yun Wang, Li Li, Hongru Jiang, Bing Zhang

**Affiliations:** National Institute for Nutrition and Health, Chinese Center for Disease Control and Prevention, Beijing, China

**Keywords:** China dietary guidelines index, Chinese adults, Diet quality, Multilevel model

## Abstract

**Background:**

The China Dietary Guidelines Index (CDGI) is a diet quality evaluation index that can present the overall diet quality and is comparable between individuals. The aim of this study was to revise CDGI for Chinese adults according to the Chinese Dietary Guidelines 2016 (CDG-2016), evaluate adherence to recommended diets between 1991 and 2015, and analyze the trend, variation, and determinants of diet quality.

**Methods:**

Food, cooking oil, and condiment intakes were estimated based on twenty-four-hour dietary recalls over three consecutive days and the household weighing method. Based on the food and nutrients recommendations for people with different energy requirements in CDG-2016, CDGI was revised as China Dietary Guidelines Index (2019)-Adults (CDGI(2019)-A) by equal weight continuity scoring. Three-level random intercept-slope growth models were applied to analyze the trend, variation, and determinants at both the community and individual levels.

**Results:**

CDGI(2019)-A, the sum of fourteen component scores with a range of 0–110 points, increased significantly from 38.2 in 1991 to 47.3 in 2015. Components with a score of less than half were milk (91.6%), fruits (72.0%), nuts (82.5%), other cereals and beans (82.6%), and seafood (77.7%). Between-individual accounted for 25.6% of the total score variation, of which 87.4% derived from the community level. CDGI(2019)-A score displayed a positive association with being female, having higher education, having higher income, living in an urban area, and knowing the CDG-2016 recommendations. The impact of income and awareness of CDG-2016 varied significantly across communities.

**Conclusions:**

Although quality of diet has been improving in China, overall quality remains poor, primarily because of inadequate intake of milk and dairy products, nuts, fruits, other cereals and miscellaneous beans, and seafood. Intervention at the community level may improve diet quality more efficiently than at the individual level, and the most effective intervention should be selected in different communities according to local conditions.

## Introduction

Optimal diet plays an important role in reducing the risk of all-cause mortality and chronic noncommunicable diseases such as hypertension, diabetes, heart disease, stroke, and colorectal cancer [1–6]. Achieving optimal diet is based on scientifically evaluating individual diet quality. Traditional evaluation of diet quality compared actual intake of foods or nutrients to the recommended intake. However, this method cannot determine overall diet quality or allow comparison of individuals’ diet quality. Evaluating overall diet quality requires establishing a comprehensive index.

Several diet quality evaluation indices exist, the earliest of which date to the 1960s. Mean adequacy ratio (MAR) and nutritional quality index (NQI) were established based on nutrient intakes [7]. Desirable dietary pattern was based on food intakes [7]. The Healthy Eating Index (HEI) [8], Alternative Healthy Eating Index (AHEI) [9], Diet Quality Index [10], Mediterranean Diet Score (MDS) [11], Dietary Approaches to Stop Hypertension (DASH) score [12], and others were based on intake of both nutrients and foods. Some of these indices have been updated with revised dietary guidelines and applied to different countries [13–21]. The main differences among these indices are their components and their methods of calculating scores. MAR was established based on recommended dietary allowances in the United States from 1968 with a full score of 100, which was the mean of multiple nutrients’ adequacy ratio. NQI includes thirty-five nutrients, and the total score ranges from 0 to 100. Although a nutrient-based dietary index is more accurate, the calculation process is complicated, and there are some limitations to its application. There are at least four MDSs as measures of adherence to the Mediterranean diet, which is a dietary pattern [11]. The more widely used algorithm is based on fifteen dietary recommendations (the Mediterranean diet pyramid) by the Mediterranean Diet Foundation, and the possible range is 0 to 15.

Based on the Chinese Dietary Guidelines 2007 (CDG-2007) and the Balanced Dietary Pagoda, we established the China Dietary Guidelines Index (CDGI) using the core method of Harvard Healthy Dietary Index. Briefly, CDGI-2007 consists of ten components: (1) coarse grains; (2) total vegetables, including the proportion of dark-colored vegetables; (3) fruits; (4) nuts, soybeans, and soybean products; (5) milk and dairy products; (6) seafood; (7) red meat and poultry; (8) edible oil; (9) salt; and (10) alcohol. Each component is scored on a continuous scale from 0 to 10. The total CDGI score has a possible range of 0–100, with a higher score indicating better compliance with the dietary guidelines. We found that adults more adherent to CDGI had a lower risk of elevated low density lipoprotein cholesterol or elevated blood glucose [22].

The Chinese Dietary Guidelines were revised in 2016, which meant that CDGI-2007 was no longer applicable. We have updated CDGI for elderly (CDGI(2019)-E) and evaluated the diet quality of the elderly from fifteen provinces, autonomous regions, and municipalities [23]. CDGI for adults has not yet been updated. Therefore, the purpose of this study is to (1) revise the CDGI for Chinese adults as CDGI(2019)-A according to the Chinese Dietary Guidelines 2016 (CDG-2016); (2) evaluate adherence to recommended diets by Chinese adults from 1991 to 2015; and (3) analyze the trend, variation, and determinants of diet quality at community and individual levels using three-level multilevel models.

### Participants and methods

#### Study sample

All data used in this study were derived from the China Health and Nutrition Survey (CHNS) codeveloped by the National Institute for Nutrition and Health of the Chinese Center for Disease Control and Prevention and the University of North Carolina at Chapel Hill in the United States [24]. CHNS was a prospective study begun in 1989 (followed up in 1991, 1993, 1997, 2000, 2004, 2006, 2009, 2011, 2015, and 2019) and was sampled by a multistage, stratified, clustered random method. The study included eight diverse provinces and autonomous regions from 1989 to 1997 and nine from 2000 to 2009. Based on the national administrative regions, a weighted sampling scheme was used to randomly select four counties and two cities in each province. Villages and townships within the counties and urban and suburban neighborhoods within the cities were selected randomly [24]. Townships and urban neighborhoods were grouped into the urban area, and villages and suburban neighborhoods were grouped into the rural area. More detailed sample and design are described elsewhere [24, 25]. Beijing, Chongqing, and Shanghai were added in 2011, and Shannxi, Yunnan, and Zhejiang provinces were added in 2015. The institutional review board of the University of North Carolina at Chapel Hill and the National Institute for Nutrition and Health, Chinese Center for Disease Control and Prevention, approved the study protocol (No. 201524). All of the participants signed the informed consents.

The data collected in 2019 are under manipulation. Adults aged 18–64 years with complete dietary, socioeconomic, and demographic data from 1991 to 2015 were involved in this study. Pregnant or lactating women, adults with energy intake less than 1600 kcal per day (kcal/d) or greater than 2400 kcal/d, and those with an implausible CDGI(2019)-A score (less than 1% of total population in each wave) were excluded. A total of 20,975 individuals living in 388 communities participated in this study. All of the participants signed the informed consents.

#### Methods of investigation

Consecutive 3d24h dietary recalls (two weekdays and one weekend day) were used in each wave to assess dietary intake at the individual level. Trained investigators interviewed each participant and then recorded the amounts and other details of all foods and drinks consumed except cooking oil and condiments. Household weighing method was used to obtain cooking oil and condiment consumption by all family members during the corresponding 3 days. The consumption of each participant was calculated according to the times cooked at home, meal proportions, and ratio of individual energy intake to household energy intake. The intake of carbohydrate and energy was calculated by means of China Food Composition [26]. The energy supply ratio of carbohydrates (%) = carbohydrates intake (g) × 4 kcal/g ÷ energy intake (kcal) × 100%. Salt intake consisted of NaCl from cooking salt, paste, and sauce.

Demographic information, income, physical activity, nutritional knowledge, and community status were all collected by trained investigators through face-to-face questionnaires. We asked the household annual income and the individual annual income of each participant each year and then calculated the household annual income per capita in different years, which were all inflated to 2015 according to the gross domestic product of each year to make them comparable. We defined whether the participants knew the CDG by their answering yes or no to this question: “Do you know the Chinese Dietary Guidelines?” The physical activity in our questionnaire had five levels: (1) very light; (2) light, meaning working in a standing position (e.g., salesperson, laboratory technician, teacher, etc.); (3) moderate (e.g., student, driver, electrician, metal worker, etc.); (4) heavy (e.g., farmer, dancer, steel worker, athlete, etc.); and (5) very heavy (e.g., loader, logger, miner, stonecutter, etc.). Body weight was measured by standard unified method and equipment. Physical activity level and body weight were used to calculate each participant’s energy requirement.

The urbanization index was established by Jones-Smith and Popkin and was based on twelve multidimensional components reflecting economic, social, demographic, and infrastructural diversity at the community level [27].

#### Energy requirement

To align with the Chinese Dietary Reference Intakes 2013, we regrouped physical activity into three levels: very light and light physical activity were considered level 1, moderate physical activity level 2, and heavy and very heavy physical activity level 3. Energy requirement was calculated by the following formula: energy requirement (kcal/d) = basal energy expenditure (kcal/kg·d) × body weight (kg) × physical activity level values. Table 1 shows basal energy expenditure by age and gender. The physical activity level values were 1.50 for level 1, 1.75 for level 2, and 2.00 for level 3 [28].

**Table 1:** Age- and gender-specific basal energy expenditure

Age (y)	Basal energy expenditure (kcal/kg·d)	
	Male	Female
18–	22.7	21.4
50–64	21.5	20.1

Source: Chinese Dietary Reference Intakes 2013

#### Revision of CDGI(2019)-a and diet quality assessment

Four of the six recommendations of CDG-2016 for the general population concerning food were selected as components of CDGI(2019)-A. CDGI(2019)-A was composed of thirteen food-related components and one nutrient-related component: 1) other cereals and miscellaneous beans, 2) total vegetables, 3) ratio of dark-colored vegetables to all vegetables, 4) fruits, 5) milk and dairy products, 6) soybeans, 7) nuts, 8) seafood, 9) poultry and meat, 10) eggs, 11) cooking oil, 12) salt, 13) alcohol, and 14) energy supply ratio of carbohydrate. “Other cereals” referred to cereals other than rice, flour, and their products. “Miscellaneous beans” referred to beans other than soybeans. “Dark-colored vegetables” referred to vegetables with carotene ≥500 μg/100 g [22]. “Energy supply ratio of carbohydrate” was used to evaluate the intake of cereals and tubers. Equivalent weight continuity scoring was used to assess the diet quality of adults in China.

The components of CDGI(2019)-A can be divided into three categories: (1) adequate intake (other cereals and miscellaneous beans, vegetables, fruits, milk and dairy products, soybeans, and nuts), (2) appropriate intake (seafood, poultry and lean meat, and eggs), and (3) limited intake (cooking oil, salt, and alcohol). For individuals with different energy requirements, neither the subtotal score of each component nor the recommended food intake amounts was equal, as detailed in Table 2. For the adequate intake foods, if intake was 0, the score was also 0; if intake was not less than the recommended amount, the score was full. For the appropriate intake foods, if intake was 0 or more than twice the recommendations, the score was 0; if intake was within the recommended ranges, the score was full. For limited intake foods, if intake was more than twice the recommendations, the score was 0; if intake was less than the recommendations, the score was full. In other cases, the score was calculated proportionally. The maximum score values were 5 for energy supply ratio of carbohydrates, 5 for other cereals and miscellaneous beans, 5 for vegetables, 5 for ratio of dark-colored vegetables to all vegetables, 10 for fruits, 10 for milk and dairy products, 5 for soybeans, 5 for nuts, 10 for seafood, 10 for poultry and lean meat, 10 for eggs, 10 for cooking oil, 10 for salt, and 10 for alcohol. The scoring method has been detailed elsewhere [23]. The total score, the sum of the fourteen components, was no more than 110 and was positive with diet quality.

**Table 2:** Components of CDGI(2019)-A and scoring

Qualitative recommendations of Chinese Dietary Guidelines 2016	Quantitative recommendations of Chinese Dietary Guidelines 2016	Components of CDGI(2019)-A	Criteria for minimum score (0)	Criteria for maximum score	Maximum score value
1. Eat a variety of foods, mainly cereals.	① Cereals and tubers 1600 kcal/d: 200 g/d 1800 kcal/d: 225 g/d 2000 kcal/d: 250 g/d 2200 kcal/d: 275 g/d 2400 kcal/d: 300 g/d	① Energy supply ratio of carbohydrates (%)	① 0% or 100%	① 50–65%	① 5
	② Whole grains and miscellaneous beans 50–150 g/d	② Other cereals (excluding rice, wheat, and tubers) and miscellaneous beans	② 0 g/d	② ≥100 g/d	② 5
2. Consume plenty of vegetables, fruits, milk, and soybeans.	① Vegetables 1600 kcal/d: 300 g/d 1800 kcal/d: 400 g/d 2000 kcal/d: 450 g/d 2400 kcal/d: 500 g/d ② Dark-colored vegetables account for half of all vegetable intake. ③ Fruits 1600 kcal/d: 200 g/d 2000 kcal/d: 300 g/d 2400 kcal/d: 350 g/d ④ Milk and dairy products 300 g/d ⑤ Soybeans 1600 kcal/d: 15 g/d 2200 kcal/d: 25 g/d ⑥ Nuts 10 g/d	① Vegetables ② Ratio of dark-colored vegetables to all vegetables ③ Fruits ④ Milk and dairy products ⑤ Soybeans ⑥ Nuts	① 0 g/d ② 0 ③ 0 g/d ④ 0 g/d ⑤ 0 g/d ⑥ 0 g/d	① 1600 kcal/d: ≥300 g/d 1800 kcal/d: ≥400 g/d 2000 kcal/d: ≥450 g/d 2400 kcal/d: ≥500 g/d ② ≥0.5 ③ 1600 kcal/d: ≥200 g/d 2000 kcal/d: ≥300 g/d 2400 kcal/d: ≥350 g/d ④ ≥300 g/d ⑤ 1600 kcal/d: ≥15 g/d 2200 kcal/d: ≥25 g/d ⑥ ≥10 g/d	① 5 ② 5 ③ 10 ④ 10 ⑤ 5 ⑥ 5
3. Consume appropriate amounts of fish, poultry, eggs, and lean meat.	① aquatic products (e.g., fish, shellfish, and mollusks) 1600 kcal/d: 40 g/d 1800 kcal/d: 50 g/d 2200 kcal/d: 75 g/d ② poultry and lean meat 1600 kcal/d: 40 g/d 1800 kcal/d: 50 g/d 2200 kcal/d: 75 g/d ③ eggs 1600 kcal/d: 40 g/d 2000 kcal/d: 50 g/d	① aquatic products ② poultry and lean meat ③ eggs	① 0 g/d or 1600 kcal/d: ≥80 g/d 1800 kcal/d: ≥100 g/d 2200 kcal/d: ≥150 g/d ② 0 g/d pr 1600 kcal/d: ≥80 g/d 1800 kcal/d: ≥100 g/d 2200 kcal/d: ≥150 g/d ③ 0 g/d or 1600 kcal/d: ≥80 g/d 2000 kcal/d: ≥100 g/d	① 1600 kcal/d: 40 g/d 1800 kcal/d: 50 g/d 2200 kcal/d: 75 g/d ② 1600 kcal/d: 40 g/d 1800 kcal/d: 50 g/d 2200 kcal/d: 75 g/d ③ 1600 kcal/d: 40 g/d 2000 kcal/d: 50 g/d	① 10 ② 10 ③ 10
4. Reduce cooking oil and salt intake, and limit sugar and alcohol intake.	① cooking oil 1600 kcal/d: 20–25 g/d 1800 kcal/d: 25 g/d 2400 kcal/d: 30 g/d ② salt < 6 g/d ③ alcohol male: ≤25 g/d female: ≤15 g/d	① cooking oil ② salt ③ alcohol	① 1600 kcal/d: ≥45 g/d 1800 kcal/d: ≥50 g/d 2400 kcal/d: ≥60 g/d ② ≥12 g/d ③ male: ≥50 g/d female: ≥30 g/d	① 1600 kcal/d: ≤20–25 g/d 1800 kcal/d: ≤25 g/d 2400 kcal/d: ≤30 g/d ② ≤6 g/d ③ male: ≤25 g/d female: ≤15 g/d	① 10 ② 10 ③ 10
		Total score			110

^a^ For participants whose energy requirement is 1600–2400 kcal/d

^b^ Dark-colored vegetables are defined as ≥500 μg carotene/100 g of vegetables

^c^ For the cooking oil, salt, and alcohol components, we chose twice the recommended maximum intake as the criteria for 0 to increase the scoring variation

#### Statistical analysis

SAS 9.4 and MLwiN 2.36 software were used in this study. Hierarchy of communities, individuals, and repeated measurements was caused by the sampling, and the CDGI(2019)-A scores (level 1) of individuals (level 2) living in the same community (level 3) for each year were not independent. Therefore, traditional analysis of variance was not applicable, so three-level multilevel models were employed in this study, implemented by MLwiN 2.36 software.

Survey year was variable at level 1. Age, gender, education, income, and awareness of the CDG were variables at individual level (level 2), of which income was in quartile. Urbanization index was variable at community level (level 3). First, a two-level null model and a three-level null model without any independent variables were fitted to analyze the intra-class correlation and variation at different levels. Then, a three-level random intercept growth model including survey years as category variate was fitted to explore the trend of total score. Subsequently, a three-level random intercept growth model, including covariates at both individual and community levels, was fitted to analyze the determinants of CDGI(2019)-A scores. Finally, a three-level random intercept-slope growth model was fitted to analyze the variation of impact of covariates on total score at the community level.

The log likelihood ratio test was used to compare the goodness of fit between models, and the Wald χ^2^ test was used to test coefficients and variances in models. Standardized residual × normal scores plots were used to test the assumption of normal distribution at different levels.

## Results

### Characteristics of the participants

Table 3 describes the characteristics of participants. In 1991 there were 5544 participants, and in 2015 there were 7458 participants. The percentage of male participants decreased from 43.6% in 1991 to 37.2% in 2015. The percentage of participants living in urban areas remained approximately consistent from 1991 to 2009, but increased significantly in 2011 and 2015. During the period of investigation from 1991 to 2015, the ages of the participants increased continuously, and the percentage of participants who knew the CDG increased significantly from 8.5% in 2004 to 29.4% in 2015.

**Table 3:** Characteristics of the participants (n, %)

	1991y	1993y	1997y	2000y	2004y	2006y	2009y	2011y	2015y
Gender									
Male	2415 (43.6)	2200 (42.1)	2093 (42.4)	2071 (39.6)	2048 (40.0)	1969 (39.9)	2011 (39.9)	2609 (39.2)	2777 (37.2)
Female	3129 (56.4)	3021 (57.9)	2837 (57.6)	3153 (60.4)	3069 (60.0)	2965 (60.1)	3031 (60.1)	4041 (60.8)	4681 (62.8)
Urban/Rural									
Urban	1832 (33.0)	1525 (29.2)	1504 (30.5)	1651 (31.6)	1599 (31.2)	1570 (31.8)	1583 (31.4)	3036 (45.6)	3060 (41.0)
Rural	3712 (67.0)	3696 (70.8)	3426 (69.5)	3573 (68.4)	3518 (68.8)	3364 (68.2)	3459 (68.6)	3614 (54.4)	4398 (59.0)
Age (y)									
18–	1831 (33.0)	1519 (29.1)	1221 (24.8)	1054 (20.2)	755 (14.8)	563 (11.4)	640 (12.7)	784 (11.8)	693 (9.3)
30–	1433 (25.8)	1346 (25.8)	1170 (23.7)	1283 (24.6)	1128 (22.0)	1012 (20.5)	902 (17.9)	1111 (16.7)	1215 (16.3)
40–	1128 (20.4)	1193 (22.8)	1310 (26.6)	1410 (27.0)	1336 (26.1)	1292 (26.2)	1295 (25.7)	1743 (26.2)	1892 (25.4)
50–	810 (14.6)	812 (15.6)	854 (17.3)	1087 (20.8)	1453 (28.4)	1523 (30.9)	1578 (31.3)	2095 (31.5)	2333 (31.3)
60–64	342 (6.2)	351 (6.7)	375 (7.6)	390 (7.5)	445 (8.7)	544 (11.0)	627 (12.4)	917 (13.8)	1325 (17.8)
Education									
≤ elementary school	2956 (53.6)	2671 (51.9)	2315 (48.0)	2098 (41.3)	1966 (38.5)	1821 (37.0)	1824 (36.2)	1895 (28.6)	1977 (26.5)
middle school	1603 (29.1)	1570 (30.5)	1457 (30.2)	1654 (32.6)	1736 (34.0)	1599 (32.5)	1771 (35.2)	2195 (33.1)	2518 (33.8)
≥ high school	955 (17.3)	903 (17.6)	1047 (21.7)	1329 (26.2)	1406 (27.5)	1501 (30.5)	1444 (28.7)	2546 (38.4)	2959 (39.7)
Income									
Q1	1389 (25.0)	1311 (25.0)	1251 (25.4)	1345 (25.8)	1310 (25.6)	1290 (26.2)	1298 (25.7)	1732 (26.0)	1950 (26.2)
Q2	1384 (25.0)	1303 (25.0)	1226 (24.9)	1291 (24.7)	1269 (24.8)	1214 (24.6)	1245 (24.7)	1639 (24.6)	1834 (24.6)
Q3	1385 (25.0)	1303 (25.0)	1226 (24.9)	1295 (24.8)	1269 (24.8)	1215 (24.6)	1252 (24.8)	1640 (24.7)	1837 (24.6)
Q4	1386 (25.0)	1304 (25.0)	1227 (24.9)	1293 (24.8)	1269 (24.8)	1216 (24.6)	1247 (24.7)	1639 (24.6)	1837 (24.6)
Urbanization index									
Q1	1365 (24.6)	1275 (24.4)	1218 (24.7)	1292 (24.7)	1263 (24.7)	1222 (24.8)	1247 (24.7)	1651 (24.8)	2003 (26.9)
Q2	1394 (25.1)	1326 (25.4)	1247 (25.3)	1315 (25.2)	1294 (25.3)	1241 (25.2)	1263 (25.0)	1673 (25.2)	1824 (24.5)
Q3	1362 (24.6)	1297 (24.8)	1213 (24.6)	1305 (25.0)	1258 (24.6)	1228 (24.9)	1240 (24.6)	1660 (25.0)	1797 (24.1)
Q4	1423 (25.7)	1323 (25.3)	1252 (25.4)	1312 (25.1)	1302 (25.4)	1243 (25.2)	1292 (25.6)	1666 (25.0)	1834 (24.6)
Chinese Dietary Guidelines									
Known	–	–	–	–	426 (8.5)	638 (13.0)	764 (15.2)	1943 (29.3)	2189 (29.4)
Unknown	–	–	–	–	4605 (91.5)	4271 (87.0)	4245 (84.8)	4691 (70.7)	5260 (70.6)
Provinces									
Original nine	5544 (100)	5221 (100)	4930 (100)	5224 (100)	5117 (100)	4934 (100)	5042 (100)	4566 (68.7)	4151 (55.7)
Beijing, Shanghai, Chongqing	–	–	–	–	–	–	–	2084 (31.3)	1553 (20.8)
Shannxi, Zhejiang, Yunnan	–	–	–	–	–	–	–	–	1754 (23.5)
Total	5544 (100)	5221 (100)	4930 (100)	5224 (100)	5117 (100)	4934 (100)	5042 (100)	6650 (100)	7458 (100)

Abbreviation: *Q* quartile

### The adherence to recommended diets in Chinese adults from 1991 to 2015

**Fig. 1:**
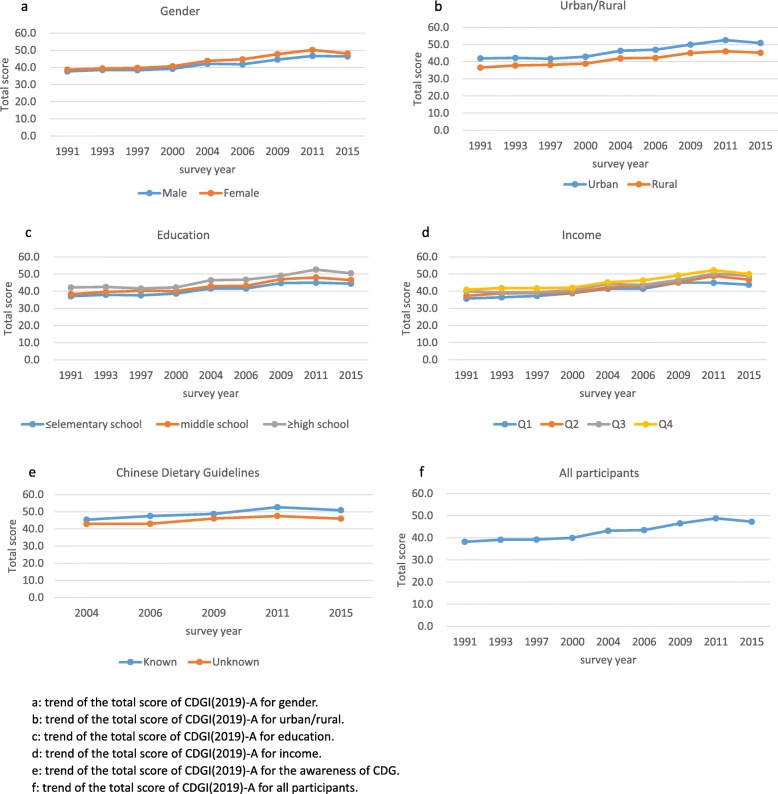
The total scores of CDGI(2019)-A of Chinese adults

Figure 1 shows the trend of the total score of CDGI(2019)-A from 1991 to 2015. For each year of the study, the CDGI(2019)-A total scores (median) were 38.2, 39.1, 39.2, 40.0, 43.2, 43.5, 46.5, 48.8, and 47.3, showing a significantly upward tendency. Females and participants living in urban areas, with higher education, with higher income, or who knew the CDG had higher total scores than males and participants living in rural areas, with lower education, with lower income, or who did not know the CDG in each year. The total score also increased with the urbanization index. Additional file 1: Table S1 shows the detailed scores.

Table 4 shows the percentage of participants in each component scored in various ranges. The percentages of CDGI(2019)-A score in the range of 0- < 55 (half of the total score) decreased from 93.7% in 1991 to 73.8% in 2015, and the range of 55- < 110 (full score) increased from 6.3% in 1991 to 26.3% in 2015. There were no participants with full score (110 points) from 1991 to 2015.

**Table 4:** Distribution of CDGI(2019)-A component scores (%)

Components	1991y			1993y			1997y			2000y			2004y		
	0-	half score-	full score	0-	half score-	full score	0-	half score-	full score	0-	half score-	full score	0-	half score-	full score
1. Energy supply ratio of carbohydrates	3.6	56.2	40.2	4.1	55.9	40.1	2.8	55.6	41.6	1.3	51.6	47.1	2.7	54.0	43.3
2. Other grains and beans	83.0	5.8	11.2	83.0	6.3	10.7	82.6	8.2	9.2	86.0	6.9	7.1	86.2	7.5	6.3
3. Vegetables	23.5	46.7	29.8	20.8	45.1	34.1	24.1	48.4	27.5	23.0	49.6	27.5	21.3	46.0	32.7
4. Ratio of deep-colored vegetables to all	58.5	18.5	23.0	59.8	19.9	20.4	51.9	21.8	26.3	56.1	20.3	23.6	54.2	25.5	20.4
5. Fruits	97.2	2.0	0.8	96.1	2.8	1.1	95.4	2.8	1.8	94.2	4.0	1.8	91.5	5.7	2.8
6. Milk and dairy products	98.7	1.1	0.2	98.8	1.0	0.2	98.8	1.0	0.2	97.6	2.2	0.3	95.0	4.3	0.6
7. Soybeans	59.8	11.0	29.2	59.3	11.7	29.1	53.2	14.8	32.0	53.1	15.9	31.0	57.1	13.4	29.5
8. Nuts	92.3	0.7	7.0	93.0	0.7	6.3	92.5	0.7	6.8	90.3	0.8	8.9	91.2	0.9	7.9
9. Seafood	83.2	14.7	2.2	82.4	15.5	2.1	82.1	16.1	1.9	80.2	17.6	2.2	81.8	16.5	1.7
10. Meat and poultry	59.9	35.9	4.3	61.5	34.4	4.1	60.9	35.7	3.4	59.5	36.5	3.9	59.1	37.2	3.7
11. Eggs	83.1	15.0	1.9	84.7	13.7	1.6	74.3	23.0	2.8	72.2	24.6	3.2	70.0	27.6	2.4
12. Cooking oil	32.3	25.2	42.5	31.2	23.1	45.6	45.5	22.4	32.1	44.5	24.2	31.4	40.9	1.7	39.3
13. Salt	76.4	11.0	12.6	70.3	11.2	18.5	70.5	14.4	15.2	71.5	16.8	11.8	51.0	20.9	28.1
14. Alcohol	5.2	0.9	94.0	5.2	0.8	94.0	5.4	0.6	94.0	4.8	0.6	94.6	4.9	0.5	94.6
Total score	93.7	6.3	0	92.7	7.3	0	92.7	7.3	0	91.3	8.7	0	84.3	15.7	0
Components	2006y			2009y			2011y				2015y				*P* *
	0-	half score-	full score	0-	half score-	full score	0-	half score-	full score		0-		half score-	full score	
1. Energy supply ratio of carbohydrates	1.9	55.2	42.9	1.6	53.1	45.3	2.4	58.7	38.9		2.1		59.4	38.5	<.0001
2. Other grains and beans	87.5	7.5	5.0	86.9	7.9	5.2	84.7	8.7	6.6		83.2		9.5	7.3	<.0001
3. Vegetables	21.5	47.4	31.2	25.1	49.5	25.4	31.8	46.1	22.1		38.0		44.5	17.5	<.0001
4. Ratio of deep-colored vegetables to all	50.6	30.2	19.3	48.0	28.1	23.9	51.7	28.3	20.0		57.0		38.6	14.4	<.0001
5. Fruits	84.9	7.9	7.2	80.0	12.5	7.5	72.0	17.0	11.0		86.9		10.1	3.0	<.0001
6. Milk and dairy products	95.6	4.1	0.3	96.6	3.0	0.4	91.6	7.0	1.5		93.9		5.3	0.8	<.0001
7. Soybeans	55.9	14.0	30.1	51.5	17.2	31.3	56.4	17.0	26.6		59.8		16.9	23.3	<.0001
8. Nuts	91.9	0.5	7.7	88.7	0.9	10.4	82.5	1.4	16.1		82.9		3.6	13.5	<.0001
9. Seafood	80.9	17.6	1.5	78.7	19.4	1.9	77.7	20.7	1.6		77.8		21.4	0.8	<.0001
10. Meat and poultry	55.0	40.2	4.8	53.2	43.0	3.8	51.7	45.3	3.0		55.7		42.9	1.4	<.0001
11. Eggs	66.9	31.2	2.0	65.5	32.5	2.0	62.9	35.2	1.9		64.3		34.7	0.9	<.0001
12. Cooking oil	45.6	23.8	30.6	43.7	24.5	31.8	40.9	24.6	34.4		38.6		21.8	39.6	<.0001
13. Salt	53.0	24.9	22.1	44.5	28.6	26.9	43.9	27.8	28.4		39.8		26.8	33.4	<.0001
14. Alcohol	6.1	0.6	93.3	5.7	0.5	94.8	1.9	0.9	97.2		1.1		0.4	98.5	<.0001
Total score	83.1	16.9	0	76.7	23.3	0	69.5	30.5	0		73.8		26.3	0	<.0001

Half score: the half scores are 2.5 points for components 1–4, 7, and 8; 5 points for components 5, 6, and 9–14; 55 points for total score

Full score: the full scores are 5 points for components 1–4, 7, and 8; 10 points for components 5, 6, and 9–14; 110 points for total score

Of the fourteen components, adherence of alcohol intake scored the best. From 1991 to 2015, the percentage of participants with full score for alcohol significantly increased from 94.0 to 98.5%. Next best was energy supply ratio of carbohydrates, with less than 4.1% of the participants having a score of less than 2.5 (full score is 5) in any year, and the percentage of participants with full score increased from 40.2% in 1991 to a peak of 47.1% in 2000 and then decreased to 38.5% in 2015. The next optimal component was cooking oil. Although the percentages of participants with full score for cooking oil decreased from 42.5% in 1991 to 39.6% in 2015, the proportion of full score was higher than for other components in each year. The percentage of participants with full score for salt rose most quickly, significantly increasing from 12.6% in 1991 to 33.4% in 2015. The percentages of participants with full score for vegetables, soybeans, and ratio of deep-colored vegetables to all vegetables declined from 1991 to 2015. The largest gaps with respect to the CDG were in intake of milk and dairy products, fruits, nuts, other cereals and miscellaneous beans, and seafood, with more than 91.6, 72.0, 82.5, 82.6, and 77.7% participants scoring less than half respectively over all study years. The low scores for these kinds of foods primarily account for the low CDGI(2019)-A total score.

### Variations and trend of CDGI(2019)-a total score

Model 1 in Table 5 shows that 25.6% of the total variation of CDGI(2019)-A score from 1991 to 2015 was between-individual and 74.4% was intra-individual. As shown in Model 2, 87.4% of the between-individual variation derived from the community level (level 3). From Model 3, we found that the CDGI(2019)-A score significantly increased 0.9, 1.4, 1.4, 4.6, 5.0, 7.5, 8.6, and 8.1 points respectively from 1993 to 2015 compared to the score in 1991.

**Table 5:** Variations and trend of CDGI(2019)-A total score

	Model 1	Model 2	Model 3
Fixed effects			
Intercept	44.177 (0.063)	45.279 (0.330)	39.758 (0.323)
Survey year			
1991	–	–	ref
1993	–	–	0.881 (0.184) ^*^
1997	–	–	1.359 (0.193) ^*^
2000	–	–	1.393 (0.189) ^*^
2004	–	–	4.571 (0.190) ^*^
2006	–	–	5.003 (0.192) ^*^
2009	–	–	7.547 (0.192) ^*^
2011	–	–	8.613 (0.190) ^*^
2015	–	–	8.114 (0.193) ^*^
Random effects			
3rd level (community)	–	40.606 (3.037) ^*^	31.734 (2.387) ^*^
2nd level (individual)	33.417 (0.823) ^*^	5.869 (0.463) ^*^	4.475 (0.415) ^*^
residual	97.273 (0.770) ^*^	96.315 (0.726) ^*^	89.492 (0.672) ^*^

Parameter estimates of fixed effect were coefficients and standard errors

Parameter estimates of random effect were variances and standard errors

Model 1 was two-level null model

Model 2 was three-level null model

Model 3 was three-level random intercept growth model

*ref* reference

^*^P < 0.001

### Impact of covariates at different levels

The coefficients of fixed effect and variances of random effect and their standard errors of the three-level multilevel models after adjusting for survey years, age, gender, education, income, urbanization index, and awareness of the CDG are presented in Table 6.

**Table 6:** Association between CDGI(2019)-A score and covariates by random intercept-slope growth models

Covariates	Three-level random intercept growth model				Three-level random intercept-slope growth model			
	Fix effect	*P*	Random effect		Fix effect	*P*	Random effect	
			3rd level (community)	2nd level (individual)			3rd level (community)	2nd level (individual)
Intercept	35.208 (0.507)	–	23.632 (1.871) *	4.230 (0.719) *	34.658 (0.509)	–	22.888 (2.632) *	3.845 (0.714) *
Survey year	0.342 (0.017)	< 0.001	–	–	0.352 (0.017)	< 0.001	–	–
Baseline age	0.004 (0.006)	0.478	–	–	0.004 (0.006)	0.490	–	–
Gender								
male	ref	–	–	–	ref	–	–	–
female	3.185 (0.129)	< 0.001	–	–	3.224 (0.138)	< 0.001	0.885 (0.464) (*P* = 0.057)	–
Education								
≤elementary school	ref	–	–	–	ref	–	–	–
middle school	1.018 (0.163)	< 0.001	–	–	1.052 (0.167)	< 0.001	0.471 (0.575) (*P* = 0.412)	–
≥high school	1.863 (0.196)	< 0.001	–	–	1.915 (0.211)	< 0.001	1.700 (0.948) (*P* = 0.073)	–
Income								
Q1	ref	–	–	–	ref	–	–	–
Q2	0.829 (0.172)	< 0.001	–	–	0.861 (0.203)	< 0.001	2.802 (0.927) (*P* = 0.002)	–
Q3	1.031 (0.181)	< 0.001	–	–	1.251 (0.252)	< 0.001	8.647 (1.571) *	–
Q4	1.245 (0.197)	< 0.001	–	–	1.436 (0.276)	< 0.001	9.924 (1.900) *	–
Urbanization								
Q1	ref	–	–	–	ref	–	–	–
Q2	0.358 (0.255)	0.160	–	–	0.497 (0.255)	0.051	–	–
Q3	1.946 (0.337)	< 0.001	–	–	2.087 (0.336)	< 0.001	–	–
Q4	2.065 (0.380)	< 0.001	–	–	2.193 (0.377)	< 0.001	–	–
Chinese Dietary Guidelines								
known	0.932 (0.169)	< 0.001	–	–	0.896 (0.226)	< 0.001	6.183 (1.221) *	–
unknown	ref	–	–	–	ref	–	–	–

Parameter estimates of fixed effect were coefficients and standard errors

Parameter estimates of random effect were variances and standard errors

Survey year: centering at 1991

ref: reference

*: *P* < 0.001

From the three-level random intercept growth model, we found that the CDGI(2019)-A score was significantly positive with the covariates mentioned previously except for age. The score significantly increased an average of 0.3 points each year, and the score of females was significantly higher than that of males by 3.2 points. Participants with middle school education scored 1.0 points higher than those with elementary school education and below, while participants with high school education and above scored 1.9 points higher than those with elementary school education and below. Compared to the lowest quartile of income, the scores of the second, third, and highest quartiles were 0.8, 1.0, and 1.2 points higher respectively. In addition, the scores of the lowest and the second quartiles of urbanization index did not differ statistically, which were about 2 points lower than both the third and the highest quartiles. The score of participants who knew the CDG was significantly higher than that of participants who did not know the CDG by 0.9 points.

From the three-level random intercept-slope growth model, we found that the impact of gender and education on the CDGI(2019)-A score was not significantly different in different communities, for the *P* values of variances were all greater than 0.05. The impact of income and awareness of the CDG on the score significantly varied in different communities, and the variances of the third and highest quartiles of income were larger than that of the second quartile in different communities.

## Discussion

We revised the CDGI-2007 for adults into CDGI(2019)-A according to the CDG-2016 by means of the equivalent weight continuity score method, which was the core of Harvard Healthy Dietary Index. Compared to the previous CDGI, the CDGI(2019)-A changed the score of other cereals from 10 points to 5 points and added two components, which were the energy supply ratio of carbohydrates for 5 points and eggs for 10 points. Therefore, the total score increased from 100 to 110. Subsequently, we calculated the CDGI(2019)-A for Chinese adults aged 18–64 years to evaluate the diet quality over a twenty-five-year period from 1991 to 2015 and further analyzed the variation and trend of CDGI(2019)-A score and its influencing factors at community and individual levels with three-level multilevel growth models. We also analyzed the components of CDGI(2019)-A score.

We found that the total CDGI(2019)-A score increased from 38.2 in 1991 to 48.8 in 2011 and decreased slightly to 47.3 in 2015, with an average increase of 0.4 per year. Despite the continuous improvement of diet quality, the score was still less than half of the full score, and diet quality can still be improved.

The dominant reason for the low total score was that the scores of milk and dairy products, fruits, nuts, other cereals and miscellaneous beans, and seafood (e.g., fish, shellfish, and mollusks) were quite low. Along with rapid economic development and social transition, China has undergone nutritional transition and dietary westernization over the past three decades. However, previous studies showed that these five kinds of foods have been seriously inadequate during this time [29–36]. Low scores of milk and dairy products, seafood, fruits, and other cereals and miscellaneous beans were also primary reasons for low diet quality among the elderly population in China [23]. Inadequate intake of these kinds of foods has become a national nutritional problem, and increasing intake should be regarded as an important goal for Chinese residents to improve their diet quality in the future. Overnutrition was also a reason for the low CDGI(2019)-A total score. For example, the average daily intake of red meat for Chinese adult males increased from 69.4 g in 1991 to 90.6 g in 2009, while daily intake for females increased from 52.2 g to 73.7 g [37].

In 2011 and 2015, we included three municipalities—Beijing, Shanghai and Chongqing—which have better adherence to recommended diets than the original nine provinces and the Shannxi, Zhejiang, and Yunnan provinces (which joined in 2015). Although including the three municipalities caused the total score in 2011 and 2015 to rise to a certain extent, it did not change the longitudinal trend of the gradual increase of the total score from 1991 to 2011, but a slight decrease in 2015. From the further analysis of the fourteen components (not shown in the results), we found that the decline of the fruit score caused by a significant decline of fruit intake from 2011 to 2015 was the primary reason for the decline of the total score from 2011 to 2015. Why the intake of fruits decreased sharply is worth exploring in depth.

The CDGI(2019)-A score in our study was not comparable with previous studies in assessing the diet quality of Chinese adults for the evaluation indices differed [38–42]. Some of those studies employed indices used abroad that were not suitable for China. The trend of diet quality in our study was not consistent with the previous study. Huang et al. found that the diet quality of Chinese residents increased from 2004 to 2006, but declined from 2006 to 2011 [41]. We found that the diet quality increased from 1991 to 2011 wave by wave, but slightly declined from 2011 to 2015. One of the possible reasons for this inconsistency was that the age of participants and exclusion criteria in the two studies were different, but the main reason was that the index components and scoring methods were completely different.

Because of the sampling method, the participants in this study had hierarchy of community-individual-repeated measurements (survey year) and aggregation at different levels. This kind of data violated the independence required by the traditional variance analysis [43]. Neglecting the hierarchy or nonindependence will result in incorrect conclusions [44]. Therefore, three-level multilevel models were used in this study.

We found that the variances of random effect at different levels showed that the impact of community level on the CDGI (2019)-A score was seven times that of individual level, suggesting that intervention at the community level might more efficiently improve the diet quality of Chinese adults than intervention at the individual level. Although we found that females and participants with higher education, with higher income, or who knew the CDG had better diet quality than males and participants with lower education, with lower income, or who did not know the CDG in each year, the degrees of impact were different at the individual level. The impact of gender on diet quality was 1.7 times that of education, 2.6 times that of income, and 3.4 times that of the awareness of CDG after adjusting for other covariates. Besides improving education, income level, and nutrition knowledge, we should focus more on males to improve diet quality. At the community level, this study only included a comprehensive index—urbanization index, which explained about 17.1% of the variation at this level. Further determinants at this level need to be identified. Income and awareness of the CDG have been determined to be variates that had significant different impact on the CDGI(2019)-A score in different communities, rather than age, gender, or education, which means that the most effective intervention should be selected in different communities according to local conditions.

Standardized residual × normal scores plots can not only test the normality of the models, but also detect the suspected outlier at different levels, which is another important characteristic of the multilevel model. This can be done in further studies.

In addition to evaluating diet quality, the diet quality evaluation index can be used as a dietary evaluation tool to study nutrition and health status. Overseas studies have found that the MDS, alternate Mediterranean Diet Score, HEI, AHEI, and DASH score are negatively correlated with the risk of cardiovascular disease incidence or death [45–48] and with the risk of colorectal cancer [49]. We previously found that CDGI-2007 score had a negative relationship with the risk of elevated low-density protein cholesterol or elevated blood glucose [22]. CDGI(2019)-A was a revision of CDGI-2007 for Chinese adults, and the impact of cardiovascular metabolic risk factors can also be analyzed theoretically. Further studies are needed to verify the association between CDGI(2019)-A and health outcomes to provide evidence for predicting the risk of chronic noncommunicable diseases.

Our study also had limitations. First, consecutive 3d24h recalls may not accurately estimate the intake of episodically consumed foods compared to nonconsecutive 24 h recalls, and this underestimated the food intakes. Second, intakes of cooking oil and salt were estimated by household weighing method. This method can represent the consumption of oil and salt only for home meals, not for meals eaten outside the home.

## Conclusions

The diet quality of Chinese adults has successively improved during the twenty-five-year period but is still relatively poor. The largest gap with respect to the food recommendations is insufficient intake of milk and dairy products, fruits, nuts, other cereals and miscellaneous beans, and seafood. Females and participants with higher education, higher income, or more awareness of CDG had better diet quality. Income and awareness of CDG had different impacts on the diet quality in different communities. Intervention at the community level may be more efficient in improving the diet quality of Chinese adults than intervention at individual level.

## Supplementary information

**Additional file 1: Table S1.** CDGI(2019)-A total score of Chinese adults aged 18–64 years.

## Abbreviations

AHEI: Alternative Healthy Eating Index
CDG: China Dietary Guidelines
CDGI: China Dietary Guidelines Index
CDGI(2019)-A: China Dietary Guidelines (2019)-Adults
CDGI(2019)-E: China Dietary Guidelines (2019)-Elderly
CHNS: China Health and Nutrition Survey
DASH score: Dietary Approaches to Stop Hypertension score
HEI: Healthy Eating Index
MDS: Mediterranean Diet Score
