# Supplementary material for: Evaluating adherence to recommended diets in adults 1991–2015: revised China dietary guidelines index
Source: Nutr J. 2019 Nov 11;18:70. doi: 10.1186/s12937-019-0498-3 (PMC6849297; doi:10.1186/s12937-019-0498-3)
Supplement: Supplementary file 1 — Additional file 1: Table S1. CDGI(2019)-A total score of Chinese adults aged 18–64 years. [file 12937_2019_498_MOESM1_ESM.docx]

**Table S1** CDGI(2019)-A total score of Chinese adults aged 18–64 years

| Items | 1991y |  |  | 1993y | |  | 1997y | |  | 2000y |  |
| --- | --- | --- | --- | --- | --- | --- | --- | --- | --- | --- | --- |
|  | mean ± sd | median |  | mean ± sd | median |  | mean ± sd | median |  | mean ± sd | median |
| Gender |  |  |  |  |  |  |  |  |  |  |  |
| Male | 38.6±9.6 | 37.7 |  | 39.1±9.8 | 38.5 |  | 39.0±9.8 | 38.4 |  | 39.8±10.0 | 39.2 |
| Female | 39.5±9.8 | 38.7 |  | 40.2±9.5 | 39.4 |  | 40.5±10.0 | 39.7 |  | 41.2±10.0 | 40.7 |
| Stratum |  |  |  |  |  |  |  |  |  |  |  |
| Urban | 42.4±10.6 | 41.9 |  | 42.8±10.3 | 42.2 |  | 42.4±10.5 | 41.7 |  | 43.4±10.6 | 42.9 |
| Rural | 37.4±8.8 | 36.6 |  | 38.5±9.0 | 37.8 |  | 38.8±9.5 | 38.2 |  | 39.4±9.4 | 38.9 |
| Age (y) |  |  |  |  |  |  |  |  |  |  |  |
| 18– | 38.7±9.6 | 37.8 |  | 39.8±9.6 | 39.3 |  | 40.2±9.6 | 40.1 |  | 40.7±9.7 | 40.5 |
| 30– | 39±9.5 | 38.2 |  | 39.7±9.6 | 38.9 |  | 40.5±10.2 | 39.8 |  | 41.0±10.3 | 40.3 |
| 40– | 39.2±10.0 | 38.5 |  | 39.8±9.6 | 39.2 |  | 39.6±9.7 | 38.4 |  | 40.8±9.8 | 40.3 |
| 50– | 39.4±9.9 | 38.4 |  | 39.7±9.8 | 38.6 |  | 39.2±10.2 | 38.3 |  | 40.1±10.1 | 39.2 |
| 60–64 | 40.7±10.3 | 39.8 |  | 39.6±9.6 | 39.1 |  | 39.2±10.6 | 38.6 |  | 40.5±10.1 | 39.5 |
| Education |  |  |  |  |  |  |  |  |  |  |  |
| ≤elementary school | 37.8±9.0 | 37.1 |  | 38.4±8.9 | 37.9 |  | 38.2±9.5 | 37.6 |  | 39.1±9.4 | 38.6 |
| middle school | 39.2±9.8 | 38.3 |  | 40.3±9.9 | 39.6 |  | 40.6±9.7 | 40.2 |  | 40.8±9.8 | 40.1 |
| ≥high school | 42.9±10.7 | 42.2 |  | 42.9±10.4 | 42.5 |  | 42.5±10.5 | 41.6 |  | 43.0±10.7 | 42.2 |
| Income |  |  |  |  |  |  |  |  |  |  |  |
| Q1 | 36.3±7.9 | 35.7 |  | 37.3±8.2 | 36.4 |  | 37.5±8.8 | 37.2 |  | 39.2±9.6 | 38.8 |
| Q2 | 37.9±9.4 | 37.3 |  | 38.8±9.1 | 38.7 |  | 39.2±9.5 | 38.9 |  | 39.6±9.4 | 39.2 |
| Q3 | 40.4±9.7 | 39.9 |  | 40.4±9.9 | 39.3 |  | 40.5±10.2 | 39.4 |  | 41.4±10.0 | 40.5 |
| Q4 | 41.7±10.8 | 40.9 |  | 42.5±10.4 | 41.8 |  | 42.3±10.6 | 41.7 |  | 42.5±10.6 | 42.0 |
| Urbanization index |  |  |  |  |  |  |  |  |  |  |  |
| Q1 | 35.6±8.0 | 35.0 |  | 36.3±7.7 | 35.5 |  | 35.4±8.4 | 35 |  | 37.6±8.7 | 37.4 |
| Q2 | 37.1±9.2 | 36.3 |  | 38.4±9.0 | 38.1 |  | 39.5±9.3 | 38.8 |  | 39.7±9.8 | 39.1 |
| Q3 | 39.6±8.9 | 39.5 |  | 39.9±9.1 | 39.5 |  | 41.1±9.7 | 40.4 |  | 41.7±9.4 | 41.3 |
| Q4 | 43.8±10.7 | 42.9 |  | 44.2±10.6 | 43.6 |  | 43.3±10.6 | 43 |  | 43.6±11.0 | 42.4 |
| Chinese Dietary Guidelines |  |  |  |  |  |  |  |  |  |  |  |
| Known | - | - |  | - | - |  | - | - |  | - | - |
| Unknown | - | - |  | - | - |  | - | - |  | - | - |
| Provinces |  |  |  |  |  |  |  |  |  |  |  |
| Original 9 | 39.1±9.7 | 38.2 |  | 39.7±9.6 | 39.1 |  | 39.9±9.9 | 39.2 |  | 40.6±10.0 | 40.0 |
| Beijing, Shanghai, Chongqing | - | - |  | - | - |  | - | - |  | - | - |
| Shannxi, Zhejiang, Yunnan | - | - |  | - | - |  | - | - |  | - | - |
| Total score | 39.1±9.7 | 38.2 |  | 39.7±9.6 | 39.1 |  | 39.9±9.9 | 39.2 |  | 40.6±10.0 | 40.0 |

**Table S1** CDGI(2019)-A total score of Chinese adults aged 18–64 years(Cont.)

| Items | 2004y |  |  | 2006y | |  | 2009y | |  | 2011y |  |  | 2015y |  |
| --- | --- | --- | --- | --- | --- | --- | --- | --- | --- | --- | --- | --- | --- | --- |
|  | mean ± sd | median |  | mean ± sd | median |  | mean ± sd | median |  | mean ± sd | median |  | mean ± sd | median |
| Gender |  |  |  |  |  |  |  |  |  |  |  |  |  |  |
| Male | 42.7±10.9 | 42.1 |  | 42.6±11.0 | 41.8 |  | 44.8±11.0 | 44.6 |  | 47.3±11.7 | 46.7 |  | 46.6±11.2 | 46.4 |
| Female | 44.4±10.9 | 43.8 |  | 45.2±11.1 | 44.7 |  | 48.0±11.1 | 47.7 |  | 50.5±12.0 | 50.2 |  | 48.4±11.3 | 48.1 |
| Stratum |  |  |  |  |  |  |  |  |  |  |  |  |  |  |
| Urban | 46.7±11.4 | 46.4 |  | 47.4±11.6 | 47.0 |  | 49.9±11.5 | 50.0 |  | 52.7±12.3 | 52.6 |  | 51.0±11.6 | 50.9 |
| Rural | 42.4±10.5 | 42.0 |  | 42.7±10.6 | 42.2 |  | 45.2±10.7 | 45.1 |  | 46.3±10.8 | 46.1 |  | 45.4±10.5 | 45.2 |
| Age (y) |  |  |  |  |  |  |  |  |  |  |  |  |  |  |
| 18– | 44.2±11.0 | 43.4 |  | 44.8±10.7 | 44.3 |  | 47.8±11.0 | 47.7 |  | 49.7±11.4 | 49.5 |  | 48.5±10.8 | 48.5 |
| 30– | 43.8±11.1 | 43.2 |  | 44.9±11.1 | 44.1 |  | 47.3±10.6 | 47.6 |  | 50.7±11.9 | 50.8 |  | 48.4±11.0 | 48.6 |
| 40– | 43.5±10.9 | 43.2 |  | 44.5±11.2 | 43.7 |  | 46.8±11.2 | 46.6 |  | 48.7±11.6 | 48.3 |  | 47.5±11.0 | 47.2 |
| 50– | 43.3±11.0 | 43.0 |  | 43.4±11.0 | 42.8 |  | 46.3±11.5 | 45.7 |  | 48.9±12.2 | 48.1 |  | 47.0±11.2 | 46.7 |
| 60–64 | 44.8±10.4 | 44.0 |  | 43.4±11.3 | 42.7 |  | 45.7±11.1 | 45.6 |  | 48.9±12.5 | 48.0 |  | 48.2±12.3 | 47.5 |
| Education |  |  |  |  |  |  |  |  |  |  |  |  |  |  |
| ≤elementary school | 42.0±10.4 | 41.6 |  | 42.0±10.3 | 41.6 |  | 44.7±10.3 | 44.7 |  | 45.4±10.8 | 45.0 |  | 44.5±10.4 | 44.4 |
| middle school | 43.4±10.6 | 42.9 |  | 43.9±10.9 | 43.1 |  | 46.7±10.9 | 47.0 |  | 48.5±11.6 | 48.0 |  | 46.9±11.1 | 46.5 |
| ≥high school | 46.6±11.5 | 46.4 |  | 47.1±11.6 | 46.7 |  | 49.3±11.9 | 49.0 |  | 52.8±12.1 | 52.6 |  | 50.5±11.4 | 50.5 |
| Income |  |  |  |  |  |  |  |  |  |  |  |  |  |  |
| Q1 | 41.8±10.2 | 41.4 |  | 41.8±9.9 | 41.4 |  | 45.1±10.1 | 45.0 |  | 45.4±11.1 | 44.9 |  | 44.3±10.5 | 43.8 |
| Q2 | 42.8±10.7 | 42.1 |  | 43.7±10.8 | 43.0 |  | 45.5±10.8 | 45.2 |  | 48.5±11.0 | 48.8 |  | 47.1±10.6 | 46.8 |
| Q3 | 44.4±10.8 | 44.1 |  | 44.6±11.5 | 43.7 |  | 46.8±11.2 | 46.5 |  | 50.8±11.9 | 50.2 |  | 48.9±11.1 | 49.0 |
| Q4 | 45.9±11.6 | 45.2 |  | 46.7±11.6 | 46.2 |  | 49.6±11.9 | 49.2 |  | 52.4±12.7 | 52.2 |  | 50.7±12.0 | 50.0 |
| Urbanization index |  |  |  |  |  |  |  |  |  |  |  |  |  |  |
| Q1 | 40.8±9.8 | 40.9 |  | 40.0±9.8 | 39.5 |  | 42.7±9.8 | 42.5 |  | 44.5±10.4 | 44.0 |  | 43.0±9.9 | 42.8 |
| Q2 | 41.2±10.4 | 40.4 |  | 42.6±10.4 | 42.6 |  | 45.8±10.6 | 45.6 |  | 47.2±11.0 | 46.9 |  | 46.5±10.6 | 46.4 |
| Q3 | 45.3±10.6 | 45.2 |  | 45.5±10.7 | 44.8 |  | 48.2±11.2 | 48.2 |  | 52.6±12.2 | 52.7 |  | 49.7±11.2 | 50.0 |
| Q4 | 47.6±11.4 | 47.7 |  | 48.5±11.6 | 48.1 |  | 50.0±11.5 | 50.2 |  | 52.6±12.1 | 52.3 |  | 52.1±11.4 | 51.7 |
| Chinese Dietary Guidelines |  |  |  |  |  |  |  |  |  |  |  |  |  |  |
| Known | 46.2±11.4 | 45.4 |  | 47.7±11.7 | 47.5 |  | 48.7±11.9 | 48.8 |  | 52.6±12.7 | 52.7 |  | 51.1±11.9 | 50.9 |
| Unknown | 43.4±10.9 | 42.9 |  | 43.6±10.9 | 43.0 |  | 46.4±11.0 | 46.1 |  | 47.8±11.4 | 47.5 |  | 46.3±10.7 | 46.0 |
| Provinces |  |  |  |  |  |  |  |  |  |  |  |  |  |  |
| Original 9 | 43.7±10.9 | 43.2 |  | 44.2±11.1 | 43.5 |  | 46.7±11.1 | 46.5 |  | 47.7±11.0 | 47.4 |  | 47.2±10.8 | 46.0 |
| Beijing, Shanghai, Chongqing | - | - |  | - | - |  | - | - |  | 52.6±13.3 | 52.6 * |  | 51.1±11.8 | 51.1 * |
| Shannxi, Zhejiang, Yunnan | - | - |  | - | - |  | - | - |  | - | - |  | 45.9±11.2 | 45.8 |
| Total score | 43.7±10.9 | 43.2 |  | 44.2±11.1 | 43.5 |  | 46.7±11.1 | 46.5 |  | 49.2±12.0 | 48.8 |  | 47.7±11.3 | 47.3 |

Abbreviation: Q=quartile.

Scores were statistically tested by multilevel models showed in Table 6.

*: CDGI(2019)-A score in Beijing, Shanghai, and Chongqing was significantly higher than the score in both the original nine provinces and Shannxi, Zhejiang, and Yunnan provinces (*P*<0.05).
